# Supplementary material for: De novo annotation reveals transcriptomic complexity across the hexaploid wheat pan-genome
Source: Nat Commun. 2025 Oct 6;16:8538. doi: 10.1038/s41467-025-64046-1 (PMC12501010; doi:10.1038/s41467-025-64046-1)
Supplement: Supplementary file 3 — Description of Additional Supplementary Files [file 41467_2025_64046_MOESM3_ESM.pdf]

Description of Additional Supplementary Files

File Name: Supplementary Data 1
Description: GO terms (Biological Process) enriched in the Core gene set

File Name: Supplementary Data 2
Description: GO terms (Biological Process) enriched in the Shell gene set

File Name: Supplementary Data 3
Description: GO terms (Biological Process) enriched in the Cloud gene set

File Name: Supplementary Data 4
Description: Summary of the number of Norin 61-specific genes.

File Name: Supplementary Data 5
Description: Summary of the total number of genes and Norin 61-specific genes in each genomic region. P-values were calculated using the two-sided Fisher's exact test.

File Name: Supplementary Data 6
Description: Location and Number of genes showing Norin 61-specific expression patterns in 176 Norin 61 unique genomic regions. P-values were calculated using the two-sided Fisher's exact test.

File Name: Supplementary Data 7
Description: Location and Number of genes showing Norin 61-specific expression patterns in 45 Norin 61 unique TE regions. P-values were calculated using the two-sided Fisher's exact test.

File Name: Supplementary Data 8
Description: List of genes showing tissue-specific expression in Norin 61. Genes written in orange, blue, and red are located on unique genomic regions, unique TE regions, and overlapping regions of unique genomic and TE regions, respectively.

File Name: Supplementary Data 9
Description: List of GO terms enriched among 107 Norin 61-specific genes with tissue-specific expression. Transposable elements were excluded from the analysis. GO enrichment analysis was performed using elim algorithm and the one-sided Fisher's exact test implemented in the topGO package with false discovery rate (FDR) < 0.05. P-values were adjusted using the Benjamini-Hochberg method.

File Name: Supplementary Data 10
Description: GO terms (Biological Process) enriched in the tandemly repeated gene set

File Name: Supplementary Data 11
Description: Module sizes and hub genes for each cultivar network

File Name: Supplementary Data 12
Description: Module membership for each cultivar network

File Name: Supplementary Data 13
Description: Metamodule membership and % shared GO terms and orthogroups (OG)

File Name: Supplementary Data 14
Description: GO term enrichment for metamodules

File Name: Supplementary Data 15
Description: Enrichment analysis of cultivar network modules for core, cloud and shell components (NS: not significant, OR: over represented, UR: under represented)

File Name: Supplementary Data 16
Description: Correlation of ME of cultivar network modules significantly enriched for cloud genes

File Name: Supplementary Data 17
Description: Top five highly connected genes for each cloud gene in Julius module JULbrown

File Name: Supplementary Data 18
Description: Network adjacency of 29 cloud genes in Julius network JULbrown module. Columns are cloud genes and rows are module members.

File Name: Supplementary Data 19
Description: Triad members split across divergent modules within cultivar networks

File Name: Supplementary Data 20
Description: GO term enrichment for divergent triads within each cultivar network

File Name: Supplementary Data 21
Description: Functional annotation of triads identified as divergent in two or more cultivars. Triads from Figure 3D highlighted in green.

File Name: Supplementary Data 22
Description: Mean expression values and log2 FC and adjusted p-values of the analysed reference allergens. Multiple test correction was performed using the Benjamini-Hochcberg (FDR) method.

File Name: Supplementary Data 23
Description: Gene set enrichment analysis of sub-genome grouped allergen protein families. Log2 FC gene expression levels were calculated for each cultivar in comparison to all other cultivars. Genes were ranked for the GSEA analysis and normalised enrichment scores (NES) values were calculated. Positive NES scores represent enrichments of the gene family in the highlighted cultivars in comparison to all other cultivars. p-values were calculated using permutation-based test with 1000 permutations.

File Name: Supplementary Data 24

Description: Coeliac disease associated epitope expression profiles. Epitope expression values are calculated using the gene expression values multiplied by the number of identfiied epitopes. Sub-genome allocations of each gene is used to collapse epitope expression levels at sub-genome level as visualised in Fig 4b.

File Name: Supplementary Data 25
Description: TFs identfied as the first neighbours of epitope containing glutenins and gliadins. Network was created using Pearson correlation value cut-off of 0.8 for co-expression.

File Name: Supplementary Data 26
Description: Gene co-expression correlation results of investigated prolamin and TF genes using Pearson correlation and two-sided t-test for p-value calculation.

File Name: Supplementary Data 27
Description: Simple Enrichment Analysis (SEA) showing Enrichment scores of TFBS detected in each epitope group. Enrichment results were filtered at 0.05 adjusted p-value with motif present in each investigated promoter sequence (100% true positives). Statistical signficiance was calculated using binomial test. Multiple test correction was performed using the Benjamni-Hochberg method.

File Name: Supplementary Data 28
Description: Identified glutenin and gliadin gene models in the pan-genome. Using the reverse translated epitope mapping approach additional glutenin and gliadin gene models have been identfiied.

File Name: Supplementary Data 29
Description: Annotated alpha gliadin gene models mapped to the locus subgraph

File Name: Supplementary Data 30
Description: Gene expression values (TPM) of chromosome 6D alpha gliadin locus gene models. Number of HLA-DQ2.5 epitopes highlighted, only gene models with complete active gene models are included.
