# Supplementary material for: De novo annotation reveals transcriptomic complexity across the hexaploid wheat pan-genome
Source: Nat Commun. 2025 Oct 6;16:8538. doi: 10.1038/s41467-025-64046-1 (PMC12501010; doi:10.1038/s41467-025-64046-1)
Supplement: Supplementary file 34 — Reporting Summary [file 41467_2025_64046_MOESM34_ESM.pdf]

nature portfolio

Corresponding author(s): Anthony Hall

Last updated by author(s): 5/8/2025

Reporting Summary

Nature Portfolio wishes to improve the reproducibility of the work that we publish. This form provides structure for consistency and transparency in reporting. For further information on Nature Portfolio policies, see our Editorial Policies and the Editorial Policy Checklist.

Statistics

For all statistical analyses, confirm that the following items are present in the figure legend, table legend, main text, or Methods section.

n/a	Confirmed	
[ ]	[x]	The exact sample size (*n*) for each experimental group/condition, given as a discrete number and unit of measurement
[ ]	[x]	A statement on whether measurements were taken from distinct samples or whether the same sample was measured repeatedly
[ ]	[x]	The statistical test(s) used AND whether they are one- or two-sided<br>*Only common tests should be described solely by name; describe more complex techniques in the Methods section.*
[x]	[ ]	A description of all covariates tested
[ ]	[x]	A description of any assumptions or corrections, such as tests of normality and adjustment for multiple comparisons
[ ]	[x]	A full description of the statistical parameters including central tendency (e.g. means) or other basic estimates (e.g. regression coefficient) AND variation (e.g. standard deviation) or associated estimates of uncertainty (e.g. confidence intervals)
[ ]	[x]	For null hypothesis testing, the test statistic (e.g. *F*, *t*, *r*) with confidence intervals, effect sizes, degrees of freedom and *P* value noted<br>*Give P values as exact values whenever suitable.*
[x]	[ ]	For Bayesian analysis, information on the choice of priors and Markov chain Monte Carlo settings
[x]	[ ]	For hierarchical and complex designs, identification of the appropriate level for tests and full reporting of outcomes
[ ]	[x]	Estimates of effect sizes (e.g. Cohen's *d*, Pearson's *r*), indicating how they were calculated

Our web collection on statistics for biologists contains articles on many of the points above.

Software and code

Policy information about availability of computer code

Data collection	No software was used to collect data for this study.
Data analysis	Many different software packages were used the data analysis for this study. All are listed and cited in the Methods section and include: HISAT2 v2.0.4, DESeq2 v1.38.3, R v3.6.0, topGO v2.50.0, triad.expression R package (https://github.com/AHallLab/triad.expression), WGCNA v1.72-1, clValid v0.7, igraph v1.4.1, ggplot2 v3.4.2, STAR v2.7.8a, StringTie v2.1.5, PacBio SMRT Link software v5.1.0.26412rev2, GMAP v2018-07-04, GenomeThreader v1.7.1, Cuffcompare v2.2.1, TransDecoder v5.5.0, Augustus v3.3.3, Mikado as implemented in the Minos pipeline (https://github.com/EI-CoreBioinformatics/minos), BLASTP ncbi-blast v2.3.0+, PTREP (Release 19; http://botserv2.uzh.ch/kelldata/trep-db/index.html), AHRD pipeline (https://github.com/groupschoof/AHRD), BUSCO v5.1.2, lastz v1.04.03, TBA/multiz, maf2hal v2.0, halLiftover v2.0, bedtools v2.30.0, JCVI, Orthofinder v2.5.1, UpSetR v1.4.0, GENESPACE v1.2.3, seaborn v0.12.1, blastn, MEME-SEA, Cytoscape, mash v2.2, PGGB & ODGI (pggb:202207121850480af7db)

For manuscripts utilizing custom algorithms or software that are central to the research but not yet described in published literature, software must be made available to editors and reviewers. We strongly encourage code deposition in a community repository (e.g. GitHub). See the Nature Portfolio guidelines for submitting code & software for further information.

Data

Policy information about availability of data

All manuscripts must include a data availability statement. This statement should provide the following information, where applicable:

- Accession codes, unique identifiers, or web links for publicly available datasets
- A description of any restrictions on data availability
- For clinical datasets or third party data, please ensure that the statement adheres to our policy

The genome sequence and gene annotations of all wheat cultivars can be viewed and downloaded in Ensembl Plants (https://plants.ensembl.org/index.html). This includes the de novo genes for the chromosome-level cultivars generated within this study and projected genes for all assemblies from the IWGSC RefSeq v1.1 annotation. All raw data used in this study is available at the European Nucleotide Archive under accession PRJEB51827.

Research involving human participants, their data, or biological material

Policy information about studies with human participants or human data. See also policy information about sex, gender (identity/presentation), and sexual orientation and race, ethnicity and racism.

Reporting on sex and gender	N/A
Reporting on race, ethnicity, or other socially relevant groupings	N/A
Population characteristics	N/A
Recruitment	N/A
Ethics oversight	N/A

Note that full information on the approval of the study protocol must also be provided in the manuscript.

Field-specific reporting

Please select the one below that is the best fit for your research. If you are not sure, read the appropriate sections before making your selection.

☒ Life sciences ☐ Behavioural & social sciences ☐ Ecological, evolutionary & environmental sciences

For a reference copy of the document with all sections, see nature.com/documents/nr-reporting-summary-flat.pdf

Life sciences study design

All studies must disclose on these points even when the disclosure is negative.

Sample size	No statistical methods were used to establish sample size. The cultivars that were sequenced were selected to represent modern breeding material representative of different geographical regions (as determined by Walkowiak et al 2020: /doi.org/10.1038/s41586-020-2961-x).
Data exclusions	Where possible we included all the RNA-seq data generated in our analyses. Data exclusion applies to only some of our subsequent analyses, where due to limitations in the data, we were unable to include them. For example, we were unable to include the five cultivars (Cadenza, Claire, Paragon, Robigus and Weebill) in identification of single copy orthogroups, as de novo annotations were not generated for these scaffolded assemblies. Any such cultivar or sample exclusions from downstream analysis are detailed in the accompanying Methods and Supplemental sections.
Replication	Each tissue was successfully sampled in triplicate from each cultivar for RNA-seq analysis, as described in the Methods section. RNA-seq libraries were successfully constructed and sequenced from samples replicated in triplicate for all six tissues (three-leaf stage (Zadoks GS13); harvesting whole roots and whole aerial organs separately, four hours after dawn (09:00), whole aerial organs two hours after dusk (23:00), complete spike at heading (GS59), flag leaf seven days post-anthesis (GS71) and whole grains 15 days post-anthesis (GS77)) for ArinaLrFor, Cadenza, Claire, Jagger, Julius, Norin61, and Weebill. As a result of RNA extraction difficulties, particularly with grain tissues, some replicates were missing as follows; LongReach Lancer (one grain sample, one aerial organs (evening) sample), Landmark (two grain samples), Mace (one aerial organs (dawn) sample, three grain samples) Stanley (one flag leaf sample, one aerial organs (evening) sample, three grain samples), SY Mattis (one grain sample), Paragon (one aerial organs (dawn) sample, three flag leaf samples) and Robigus (one grain sample, one root sample).
Randomization	Randomization does not directly apply to the sequencing carried out in this study, however where randomisation was part of downstream computational analysis (such as data seeding or bootstrapping) it has been used as described in the Methods section.
Blinding	This study focuses on transcriptomics and genomic analysis and as such did not involve blinding.

Reporting for specific materials, systems and methods

We require information from authors about some types of materials, experimental systems and methods used in many studies. Here, indicate whether each material, system or method listed is relevant to your study. If you are not sure if a list item applies to your research, read the appropriate section before selecting a response.

Materials & experimental systems

n/a	Involved in the study
☒	☐ Antibodies
☒	☐ Eukaryotic cell lines
☒	☐ Palaeontology and archaeology
☒	☐ Animals and other organisms
☒	☐ Clinical data
☒	☐ Dual use research of concern
☐	☒ Plants

Methods

n/a	Involved in the study
☒	☐ ChIP-seq
☒	☐ Flow cytometry
☒	☐ MRI-based neuroimaging

Plants

Seed stocks

Seed obtained from Germplasm Resources Unit (JIC, Norwich UK) https://www.seedstor.ac.uk/ Lines used: Mace: PANG0011, LongReach Lancer: PANG0009, CDC Stanley: PANG0004, CDC Landmark: PANG0003, Julius: PANG0007, Norin 61: PANG0012, ArinaLrFor: PANG0001, Jagger: PANG0006, SY Mattis: PANG0015, Cadenza: PANG0002, Claire: PANG0005, Paragon: PANG0013, Robigus: PANG0014, Weebill: PANG0016

Novel plant genotypes

N/A

Authentication

N/A
